# Commencements—Dental Colleges

**Published:** 1897-04

**Authors:** 


### Commencements—Dental Colleges.

The time is near at hand when the dental colleges, or some of them at least, close the year's work; when the teachers will sum up the results of their labor, as shown in the classes with which they have had to do. There is hardly anything more gratifying than a realization of work well done; that is satisfactory to both teacher and pupil. Such a gratification as shall be a stimulus for future effort that is really a preparation for that which is to be taken up again in the not distant future, and especially for those whose preparatory work is not completed. For those who are to go out, with the indorsement of their alma mater, an uncertain future lies before them, more or less pronounced it may be, nevertheless uncertain, but susceptible of being wrought out into certainty, if there is a reasonable amount of preparation in the way of professional attainment, industry, perseverance, discrimination and will power. With these all in right balance and properly used success is well nigh assured.

There is oftimes much misgiving on the part of those who are about to enter an untried field, and especially if it is to be a life-work and one involving prosperity or adversity. The question: "Shall I be a failure?" too often forces itself upon the mind of the beginner in life's work. Pondering such questions can serve no desirable purpose, so dismiss them at once, and cultivate a determination to succeed. There is an ample field, especially for fully equipped dentists; yes, a loud call for all such. Then, let every one prepare himself to occupy an honorable and useful position.

There will be about sixteen hundred graduates from all the reputable dental colleges of the United States at the close of the present college year. The number is increasing from year to year, in 1896 it was fourteen hundred and forty-six, and judging from the growth of former years the number indicated above is not unreasonable.

There is at the close of each year solicitude expressed as to what is to become of this large number of practitioners. Is it at all likely that there is, or will be a demand, or even an invitation for the exercise of so much talent of this sort? This fear is based on the supposition that the teeth of the people of the country have all the attention they need. This is a supposition not at all in accordance with the facts, as intelligent, general and persistent observation will fully verify. A practitioner of long experience and eminent ability has often declared in public speech, as well as private, that there are not enough well-equipped dentists in the United States to keep the teeth of all the people clean. This statement, while it may at first view seem extravagant, a little deliberate thought will convince almost any one that it is not very wide of the truth.

Of the whole number of graduates from our colleges, at least ten percent will drop out in three years; fifteen percent will not attain reasonable success; sixty percent will attain a reasonable success, while twenty percent will attain good success, and this means efficient practice, and good business ability. A very reasonable estimate gives the annual loss in the dental profession, by death, at four hundred, and dropping out of the profession for other reasons, at two hundred and fifty. By these two influences six hundred and fifty vacancies are made, and certainly not less than seven hundred additional dentists are required to keep up with the growth of population; and, furthermore, in making these estimates it must not be forgotten that there are thousands of communities that either have no dentist at all or are poorly supplied in number or quality, or both. Now, in view of these things, is there any probability that there will be, in the next twenty-five years, an excess of well-qualified dental practitioners?

The demand for thoroughly-qualified dentists is very great; for those of medium qualifications the demand is very moderate, and for those of inferior qualifications there is no demand.

